# Modulation of IRAK4 as a Therapeutic Strategy Against Monosodium Urate- and Xanthine-Induced Inflammation in Macrophages and HepG2 Cells

**DOI:** 10.21203/rs.3.rs-6908346/v1

**Published:** 2025-07-16

**Authors:** Sadiq Umar, Huan T Chang, Mark Maienschein-Cline, Sriram Ravindran

**Affiliations:** University of Illinois Chicago; Jesse Brown VA Medical Center; University of Illinois at Chicago; University of Illinois Chicago

**Keywords:** IRAK4 inhibitor, macrophages, HepG2, inflammation, NF-κB signaling

## Abstract

**Background:**

Interleukin-1 receptor-associated kinase 4 (IRAK4) is a pivotal mediator of toll-like receptor (TLR) and interleukin-1 receptor (IL-1R) signaling, critically involved in innate immune activation and pro-inflammatory cytokine production. Dysregulated IRAK4 activity contributes to chronic inflammation in both immune and non-immune cells. In this study, we evaluated the immunomodulatory potential of a selective IRAK4 inhibitor on monosodium urate (MSU) crystals-stimulated macrophages and xanthine-challenged HepG2 cells to assess its therapeutic potential.

**Methods:**

Human PBMCs were pretreated with 1 μM IRAK4 inhibitor (IRAK4i) overnight, followed by stimulation with 100 μg/ml MSU for either 30 minutes or 24 hours. Conditioned medium was collected for ELISA and RNA for qPCR to quantify pro- and anti-inflammatory factors. Cell lysates were prepared to analyze various TLR/IL-1β signaling proteins, including phosphorylated IRAK4, P38, ERK, and JNK. Phagocytosis was assessed using a Vybrant^™^ phagocytosis assay kit in PBMCs. We also utilize HepG2 cells and pretreated with 1 μM IRAK4 inhibitor (IRAK4i) overnight, followed by stimulation with 2.5mM of xanthine for 24 hours to assess the expression of cytokine and xanthine oxidoreductase.

**Results:**

Primary macrophages and HepG2 cells were treated with a potent IRAK4 inhibitor in the presence and absence of MSU or xanthine. In macrophages, IRAK4 inhibition significantly reduced the expression of TNF-α, IL-6, and IL-1β at both mRNA and protein levels, while promoting polarization toward an anti-inflammatory (M2-like) phenotype alongside reduced activation of NF-κB and MAPK pathways. In HepG2 cells, IRAK4 blockade attenuated xanthine-induced expression of xanthine dehydrogenase and inflammatory cytokines.

**Conclusion:**

These findings demonstrate the dual anti-inflammatory effect of IRAK4 inhibition in both immune and hepatic cells and suggest a promising strategy to mitigate inflammation in gout.

## Introduction

Gout is presently recognized as common root of inflammatory arthritis. Its incidence and prevalence are on the rise in both developed and developing nations^1–4^. The inflammatory response in gout is triggered by the deposition of uric acid crystals in the articular joints of individuals with hyperuricemia, leading to severe inflammation and excruciating pain. In the body, purine metabolism produces uric acid as a byproduct, and its imbalance between production and excretion or reabsorption results in elevated levels of uric acid in bodily fluids^5–7^. The incidence of gout is further increased in individuals who consume red meat and alcohol^8^. Uric acid deposition can also lead to complications such as renal dysfunction, cardiovascular diseases, and diabetes^9–12^.

Research has shown that the initial inflammatory response in gout involves articular resident macrophages within the joint space phagocytosing monosodium urate (MSU) crystals. These engulfed MSU crystals interact with Toll-like receptors (TLR) especially TLR2/4^13–16^. These TLRs’ recognition of MSU leads to the activation of nuclear factor-κB (NF-κB) and the NLRP3 inflammasome, resulting in the activation of caspase-1 and the processing and secretion of interleukin-1β (IL-1β). IL-1β, along with other pro-inflammatory cytokines such as IL-6, TNF-α, and IL-8, promotes neutrophil invasion^17–22^. Infiltration of these neutrophils contribute to joint damage through the release of various mediators, including reactive oxygen species, cytokines, chemokines, proteolytic enzymes, and prostaglandin E2 (PGE2), which leads to degradation of cartilage ^23–25^.

Interleukin-1 receptor-associated kinase 4 (IRAK4) is a serine-threonine kinase in the TLR/IL-1R signaling cascade^26^. Upon stimulation, the cytoplasmic receptor domain binds to the intracellular adaptor protein MyD88, resulting in the assembly of MyD88 and IRAK family (IRAKs 1–4) into a complex known as the Myddosome. IRAK4 initiates the production of pro-inflammatory cytokines via the activation of transcription factors (NF-κB and AP-1)^5, 13, 24, 27–29^. Our studies have shown that the onset of rheumatoid arthritis (RA) is abrogated by the inhibition of IRAK4 in TLR7-induced inflammation in macrophage and fibroblast cell-based and in vivo models of joint inflammation^30–32^.

Previous studies have shown that TLR2/4 activation amplifies inflammation in myeloid cells through NF-κB signaling and expression of these receptors correlates with disease activity in gout^33^. Notably, TLR2/4 levels decrease in patients during remission compared to active flare^34^. Given this pattern, we explored whether targeting IRAK4—a common downstream mediator of TLR signaling—could attenuate the inflammatory response associated with gout. Our findings support this approach, showing that IRAK4 blockade dampens key inflammatory pathways activated by both MSU and xanthine.

## Results

### TLRs and cytokines associated with gout flare and remission.

Toll-like receptors (TLRs) contribute to the initiation and progression of inflammation in arthritic diseases by recognizing harmful stimuli and activating immune pathways that lead to the production of pro-inflammatory cytokines and mediators. To explore the role of TLRs and key cytokines in gout pathogenesis, we reanalyzed a publicly available dataset^34^, focusing on the expression of TLRs, IL-1β, IL-18, TNF-α, IL-10, and TGF-β in patients experiencing gout flare versus remission.

**Figure 1:**
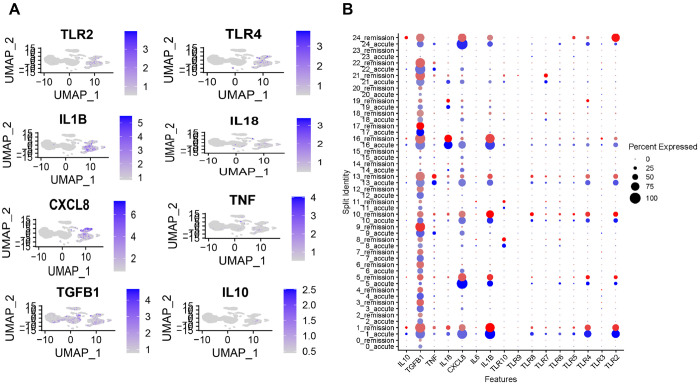
TLR2 and TLR4 are associated with the severity of gout. **(A)** UMAP plot showing distinct clustering of cell populations in patients with gout flare and remission phase (**B**) Expression levels of TLRs and cytokines in gout flare and remission. Dot plot represented by color gradient, with gout flare depicted by blue and remission shown in red.

Our analysis revealed that TLR2 and TLR4 expression was significantly reduced during remission compared to the flared state, whereas other TLRs exhibited similar trends without substantial changes (Fig. 1A and B). Consistent with reduced TLR activation, the expression of IL-1β and IL-18 key inflammatory cytokines of the IL-1 family—also decreased during remission, while TGF-β, an anti-inflammatory cytokine, was upregulated. Together, these findings suggest that TLR2 and TLR4 are key drivers of IL-1 family cytokine signaling in gout and may play a central role in disease exacerbation.

### Inhibition of IRAK4 disrupts MSU-induced cytokine production.

**Figure 2:**
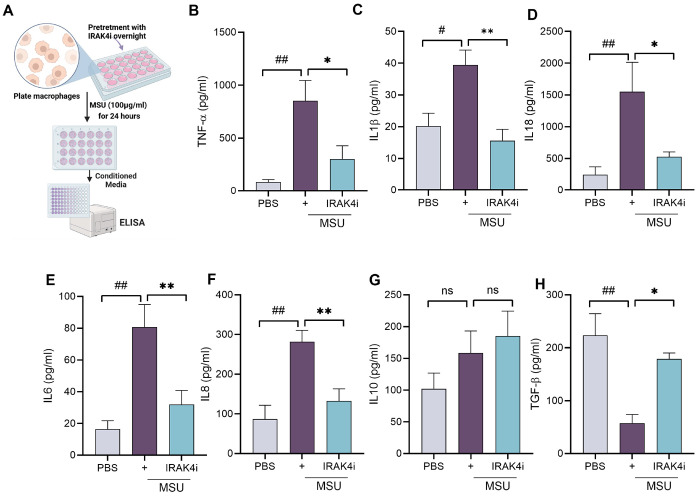
IRAK4 inhibition reduces MSU-induced pro-inflammatory cytokine production in human macrophages (**A**) schematic diagram to show experimental design from isolation of monocytes from PBMC and its differential into macrophages and pretreatment with IRAK4i (1μM/ml) overnight and stimulation with MSU (100 μg/ml) for 24 hrs, (**B-H**) Conditioned media was utilized for quantifying cytokines such as IL-1β, TNF-α, IL-6, IL8, IL18, IL10 and TGF-β secretion by ELISA. n=5-6. The data are shown as mean ± SEM, ^#^ represents p<0.05 and ^##^ denotes p<0.01 as compared to PBS, * represents p<0.05 and ** denotes p<0.01 as compared to MSU. Significant differences were determined by one-way ANOVA following Šídák’s multiple comparison test.

As TLRs play a role in gout, as shown in Fig. 1, targeting downstream components of TLR signaling presents a promising therapeutic strategy. IRAK4, a key kinase in the TLR/IL-1 receptor pathway, mediates the activation of NF-κB and MAPKs, leading to the production of pro-inflammatory cytokines such as IL-1β and IL-18. We investigated the inflammatory response triggered by monosodium urate (MSU) crystals in human monocyte-derived macrophages and delineated the mechanism by which IRAK4 inhibition modulates this response. Notably, MSU exposure led to a robust increase in IL-18 secretion, followed by elevated levels of TNF-α, IL-6, IL-1β, and IL-8 (Fig. 2B–F) and decrease in TGF-β and no change in IL10. Treatment with an IRAK4 inhibitor (IRAK4i) significantly attenuated MSU-induced cytokine production by approximately 50–70% and raise TGF-β by 70%.

**Figure 3:**
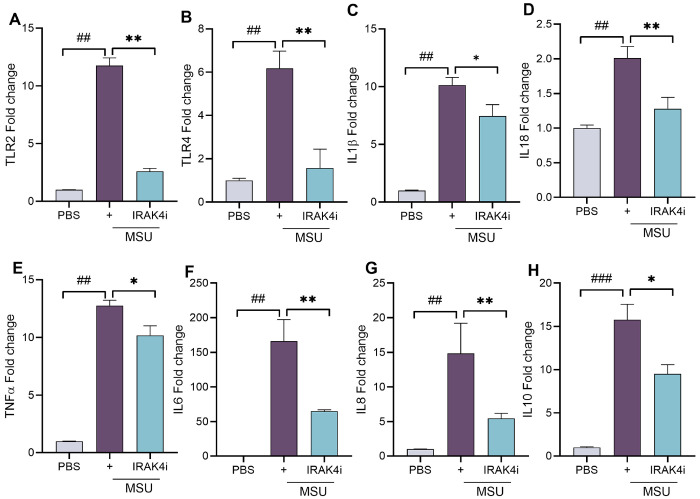
IRAK4 inhibition attenuates MSU-induced expression of inflammatory genes in human macrophages (A-H) Monocytes were isolated from PBMC and differentiated into macrophage with similar experimental conditioned as stated in fig 2A. Cells were harvested for RNA isolation and mRNA expression of as TLRs(TLR2 and TLR4) and cytokines (IL-1β, TNF-α, IL-6, IL8, IL18, and IL10) by real-time RT-PCR, n=5-6. The data are shown as mean ± SEM, ^#^ represents p<0.05 and ^##^ denotes p<0.01 as compared to PBS, * represents p<0.05 and ** denotes p<0.01 as compared to MSU. Significant differences were determined by one-way ANOVA following Šídák’s multiple comparison test.

At the transcriptional level, IRAK4i markedly suppressed the expression of TLR2 and TLR4, reducing their induction by 70–80% (Fig. 3A–B). Furthermore, MSU stimulation strongly upregulated IL-1β, TNF-α, IL-8, IL-18 (~ 10–15 fold), and IL-6 (~ 150-fold), all of which were significantly suppressed (by 40–60%) following IRAK4 inhibition (Fig. 3C–G). We also observed an increase in IL-10 levels following MSU stimulation, which may represent a compensatory response by macrophages to counteract the inflammatory environment. Collectively, these findings demonstrate that IRAK4i effectively reverses the MSU-induced inflammatory phenotype, primarily by suppressing TLR signaling in human monocyte-derived macrophage.

### Inhibition of IRAK4 disrupts MSU-mediated phagocytic activity and suppresses NF-κB

**Figure 4:**
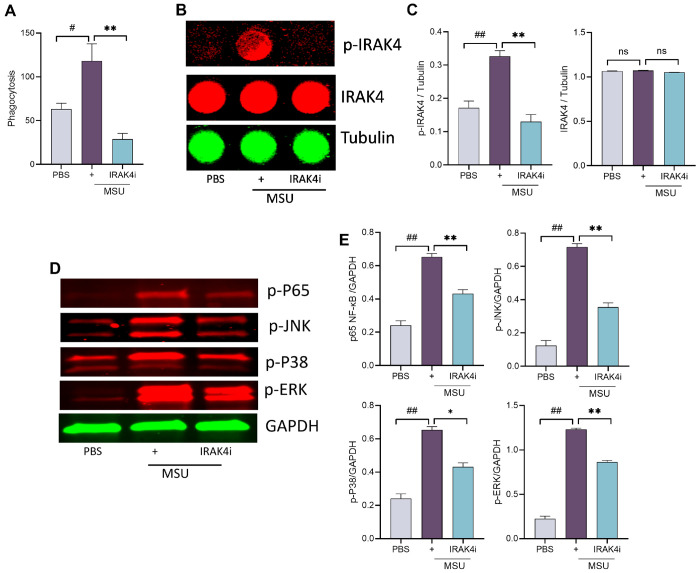
IRAK4 inhibition impairs MSU induced phagocytosis and suppresses NF-κB signaling. **(A)** macrophages were seeded in to 96 well plate and incubated overnight with IRAK4i (1 μM/ml) and stimulated with MSU (100 μg/ml) for 2 hr. and then follow manufacturer instructions. **(B-C)** For in cell western, macrophages were seeded in to 96 well plate (black) and incubated overnight with IRAK4i (1 μM/ml) and stimulated with MSU (100 μg/ml) for 30 min, cell were fixed, blocked, and incubated with primary (p-IRAK4, IRAK4, Tubulin, 1:200) and secondary antibody. Images were taken in licor and analyzed. **(D-E)** macrophages were seeded in to 6 well plate and incubated with IRAK4i and stimulated with MSU (100 μg/ml) for 30 min. Lysates were probed for p-ERK, p-p38, p-JNK, and p-NF-kB(p65) (1:1000, Cell signaling) and normalized to GAPDH (1:3000, Cell signaling), n=3. Western blot density was analyzed by Image J. The data are shown as mean ± SEM, ^#^ represents p<0.05 and ^##^ denotes p<0.01 as compared to PBS, * represents p<0.05 and ** denotes p<0.01 as compared to MSU. Significant differences were determined by one-way ANOVA following Šídák’s multiple comparison test.

Phagocytosis is a key driver of inflammation in gout. Upon engulfing monosodium urate (MSU) crystals, macrophages activate TLR and downstream signaling pathways, leading to the release of pro-inflammatory cytokines such as IL-1β and IL-18 and amplifying inflammation via NF-κB activation. Our results demonstrated that IRAK4 inhibition effectively blocked MSU-induced phagocytosis in PBMCs (Fig. 4A).

To further investigate the mechanism, we examined whether IRAK4i affects IRAK4 phosphorylation and downstream NF-κB signaling. Exposure of human myeloid cells to MSU activated IRAK4 and led to phosphorylation of MAPKs (p38, ERK, and JNK), culminating in NF-κB pathway activation (Fig. 4B–E, Suppl. Figure 1). Notably, IRAK4i suppressed MSU-induced activation of both IRAK4 and MAPK signaling in these cells. These findings suggest that targeting phagocytosis and its downstream effectors, particularly IRAK4, is an effective strategy to interrupt the inflammatory cascade triggered by MSU.

### IRAK4 Inhibitor Attenuates Xanthine-Stimulated Cytokine Response in HepG2 Cells

In gout, dysregulation of the uric acid cycle can negatively impact liver as it is a major site of purine metabolism, where xanthine oxidoreductase converts hypoxanthine and xanthine into uric acid. Moreover, elevated xanthine and uric acid levels can stimulate cytokine production in hepatocytes (e.g., HepG2 cells). Thus, in gout, the overactive uric acid cycle may burden the liver with both metabolic and inflammatory stress, exacerbating disease beyond the joints.

We further tested if IRAK4i have any benefits in suppressing the inflammation induced by xanthine in liver. We took HepG2 cells and activate them with different dose of xanthine (0-2.5mM) and look at the expression of XDH. Our results showed increase of XDH expression dose dependently. Further we treated HepG2 with IRAK4i (1μM) overnight and stimulated with xanthine for 24 hr. The expression of XDH and cytokines were increase while IRAK4i inhibits their expression significantly, which showed the effect of IRAK4i not only on macrophages but on HepG2 cells too.

## Discussion

Over the past decade, multiple studies—including our own—have underscored the critical role of IRAK4 in regulating inflammation. While much of the research has focused on its involvement in conditions such as rheumatoid arthritis^30 32, 35–37^, psoriatic arthritis^31, 38^, COVID-19^39–43^, epilepsy^44^, acute myeloid leukemia^45^, and acute lung injury^46–48^, its role in gout has remained largely unexplored. This study addresses that gap by investigating the effect of IRAK4 inhibition in an in vitro gout model. Our findings show that IRAK4 is a key mediator of inflammation triggered by MSU. Inhibition of IRAK4 significantly suppressed MSU-induced inflammatory signaling via the TLR–NF-κB pathway, while also reducing xanthine-induced cytokine production and XDH expression in HepG2 cells. These results highlight IRAK4’s central role in gout-associated inflammation and highlighting its broader anti-inflammatory potential beyond immune cells.

Prior studies in knockout mouse models have demonstrated that TLR2 and TLR4 are key receptors recognizing MSU crystals^18, 20, 33, 49^. Consistent with these findings, we observed that MSU stimulation increased TLR2 and TLR4 transcription in human PBMCs, while treatment with IRAK4i effectively suppressed their upregulation, suggesting that IRAK4 inhibition interferes with MSU-driven TLR signaling. IRAK4 inhibition significantly attenuated the MSU-induced inflammatory response, as evidenced by reduced expression of IL-1β, IL-18, IL-6, IL-8, and TNFα at both the transcriptional and protein levels.

Uric acid, a common cellular metabolite, in its crystalline form as monosodium urate (MSU) is released from dying or damaged cells^50^. The enhanced phagocytosis of MSU crystals is strongly linked to increased gout severity, as their uptake by neutrophils and macrophages triggers a robust inflammatory response^51–54^. Notably, IRAK4 inhibition markedly suppressed MSU-induced phagocytosis in human macrophages, indicating its role in dampening early inflammatory triggers. To further elucidate the underlying mechanisms, we examined the phosphorylation status of key proteins in the NF-κB signaling pathway. Our results showed that IRAK4i significantly reduced the phosphorylation of these signaling molecules, thereby downregulating NF-κB pathway activity and limiting the downstream pro-inflammatory response in macrophages.

Given the liver’s central role in purine metabolism and uric acid production, and to evaluate the broader anti-inflammatory effects of IRAK4 inhibition beyond immune cells, we utilized HepG2 hepatocyte-like cells as an in vitro model. Earlier studies showed the activation of IRAKs in HepG2 treated with palmitic acid^55^. HepG2 cells were stimulated with xanthine to mimic metabolic stress observed in gout^56, 57^. Our findings showed that xanthine stimulation upregulated XDH and pro-inflammatory cytokines, whereas IRAK4 inhibitor treatment significantly suppressed both XDH expression and cytokine production, indicating that IRAK4 signaling may also contribute to liver inflammation in the context of gout. These results suggest a potential role for IRAK4 inhibition in mitigating systemic and hepatic inflammatory responses linked to uric acid dysregulation.

Although clinical trials have demonstrated the therapeutic potential of IL-1 inhibitors like anakinra and more recently rilonacept, their limitations in effectively managing gout have also been recognized^58–61^. These challenges highlight the need for novel, targeted therapies that not only address current shortcomings but also offer more practical delivery options. Our study demonstrates that inhibition of IRAK4 signaling significantly reverses the pro-inflammatory profile induced by MSU crystals in human monocyte-derived macrophages. By disrupting key components of the TLR–NF-κB signaling axis, IRAK4 inhibition reduces the expression of critical cytokines such as IL-1β, IL-18, IL-6, IL-8, and TNFα. These findings not only reinforce the central role of IRAK4 in mediating gout-associated inflammation but also suggest that pharmacological targeting of IRAK4 may offer a promising therapeutic strategy to control excessive inflammation in patients with gout. Future experiments will focus on animal models of gout where different delivery mechanisms at site-specific and systemic levels will be evaluated for dose response, efficacy and toxicity.

## Methods

### Gene expression analysis from gout patients:

The single-cell RNA sequencing dataset GSE211783 submitted by Hanjie Yu et al.^34^ for gout flare and gout remission was accessed via the web interface (https://www.ncbi.nlm.nih.gov/geo/query/acc.cgi?acc=GSE211783) to evaluate the expression of TLRs and cytokines. This cohort consisted of gout patients who were older than 18 years and meeting the 2015 ACR/EULAR classification criteria.

## Cell culture:

### Human myeloid cells:

The study was approved (approval number: 2021 – 1435) by Institutional Ethics Review Board, University of Illinois at Chicago (UIC). Healthy samples were purchased from (Oklahoma Blood Institute, Oklahoma City, OK). Peripheral blood mononuclear cells (PBMCs) were isolated from healthy donors using Ficoll Paque based density centrifugation, as described earlier^30^. Human monocytes from healthy donors were differentiated into macrophages (MΦs) over three days in RPMI medium containing 10% FBS. On day 4, MΦs were pretreated for 18 hr with DMSO (PBS), IRAK4i (1 μM, Sigma #PZ0327)^30^ in serum free RPMI. Thereafter cells were stimulated with MSU^29^ (100 μg/ml; Sigma #U2875) for 24 hr. for running ELISA (Protein) and qRT-PCR (mRNA) analysis.

### HepG2 Cells

HepG2 was generously provided by Khetani Lab, Department of Biomedical Engineering, University of Illinois. HepG2 cells were cultured in 10% DMEM. Confluent cells were seeded in 24 well plate and pretreated for 18 hr with DMSO (PBS), IRAK4i (1 μM, PF06650833, Sigma #PZ0327) in DMEM (without FBS). Thereafter cells were stimulated with Xanthine (2.5 mM; Sigma # X7375)^62^ for 24hr for mRNA analysis.

### Real-time RT-PCR

RNA isolated using Trizol and was reverse transcribed to cDNA using the RevertAid RT Reverse Transcription Kit (Thermo Scientific). SYBR green gene expression master mix (Bio-Rad) to perform qRT-PCR. Data was normalized with GAPDH and are presented as fold changes in RNA levels compared to control treatment, calculated following the 2 – ΔΔCt method.

### ELISA for cytokine analyses

Conditioned media from the macrophage, pretreated with IRAK4i (1μM/ml) overnight followed by stimulation with MSU for 24 hr was collected and cytokine levels of IL-1β, IL-6, IL-8, IL-10, TNF-α, IL-18 and TGF-β were measured using DuoSet ELISA (enzyme-linked immunosorbent assay) kits (R&D Systems, MN).

### In vitro phagocytosis assay

The phagocytic activity of macrophages was assessed using the Vybrant^™^ Phagocytosis Assay Kit (Life Technologies^™^). Briefly, macrophages (1×10^4^) were seeded in a 96-well flat-bottom plate, pretreated with IRAK4i overnight, and stimulated with MSU for 2 hours. The culture medium was then replaced with 100 μL of the prepared fluorescent Bioparticle suspension, followed by incubation at 37°C for 2 hours. After incubation, the Bioparticle suspension was removed, and the cells were washed twice with PBS. Subsequently, 100 μL of prepared Trypan Blue suspension was added, incubated for 1 minute, and the fluorescence intensity was measured using a plate reader with ~ 480 nm excitation and ~ 520 nm emission, following the manufacturer’s instructions.

#### Immunoblotting:

Human macrophages were pretreated with IRAK4 inhibitor, next day cells were stimulated with MSU (100 μg/ml; Sigma #U2875) for 30 min. Cells were lysed in RIPA buffer and probed for p38, JNK, ERK and NF-kB phosphorylation, (1:1000, Cell Signaling) and GAPDH equal loading (1:3000, Santa Cruz).

### In-Cell Western

ICW was performed to quantify the relative levels of phosphorylation of IRAK4 in PBMC following treatment as mentioned earlier. Briefly, macrophages were seeded in 96- well plates at a density of (1×10^4^) cells per well. Cells were then pretreated with IRAK4 inhibitor overnight and stimulated with MSU for 30 min. Following treatment, cells were fixed with 4% paraformaldehyde and permeabilized using 0.1% Triton X-100. Cells was blocked and incubated with primary antibodies against p-IRAK4, IRAK4 and tubulin overnight at 4°. After washing with PBST, cells were incubated with IRDye-labeled secondary antibodies (LI-COR) for 1hr at room temperature in the dark. Fluorescent signal was detected using an Odyssey CLx Imaging System (LI-COR).

### Statistical Analysis

For comparison between multiple groups, one-way ANOVA followed by Tukey’s multiple comparison test was done using Graph Pad Prism10 software. Values of *p* < 0.05 were considered significant.

## Supplementary Material

This is a list of supplementary files associated with this preprint. Click to download.

- Supplementary.docx

## Figures and Tables

**Figure 5:**
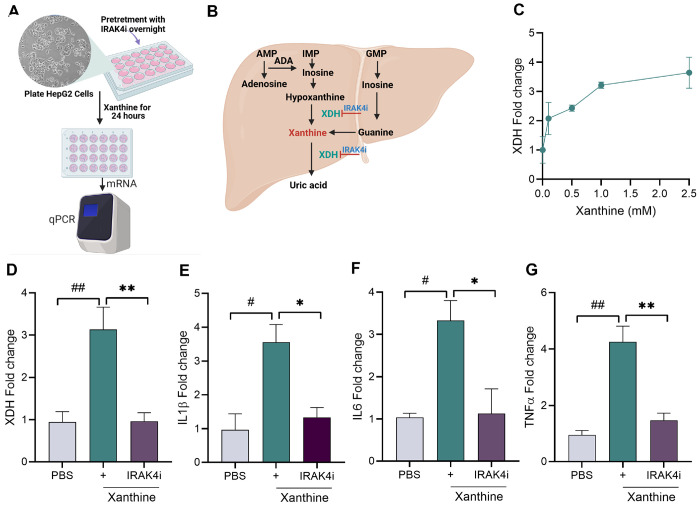
IRAK4 inhibition reduces xanthine-induced XDH and pro-inflammatory cytokine expression in HepG2 cells **(A-C)** schematic diagram to show experimental design, HepG2 cells were seeded in 24 well plate and stimulated with xanthine (0-2.5 mM), expression of XDH was evaluated by real-time RT-PCR. **(D-G)** HepG2 cells were seeded in 24 well plate and pretreatment with IRAK4i (1μM/ml) overnight and stimulation with xanthine (2.5 mM-based on dose dependent study) for 24 hrs., Cells were harvested for RNA isolation and mRNA expression of as XDH and cytokines (IL-1β, TNF-α, and IL-6) by real-time RT-PCR, n=3. The data are shown as mean ± SEM, ^#^ represents p<0.05 and ^##^ denotes p<0.01 as compared to PBS, * represents p<0.05 and ** denotes p<0.01 as compared to xanthine. Significant differences were determined by one-way ANOVA following Šídák’s multiple comparison test.

**Figure 6:**
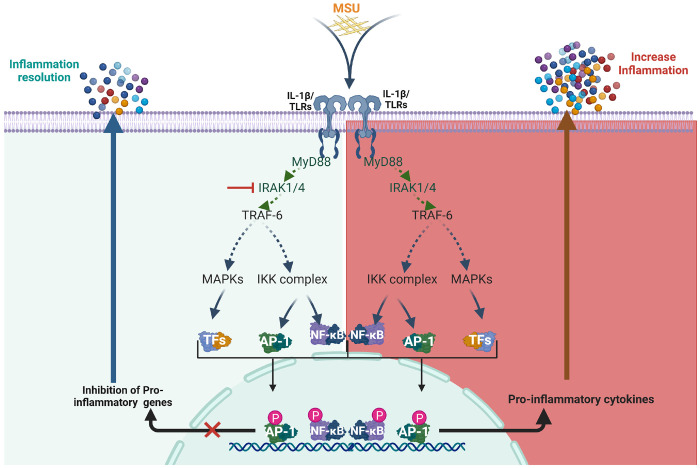
Schematic representation of the mechanism by which IRAK4 inhibition modulates MSU-induced inflammation.

## Data Availability

All findings are exhibited in the paper and the material and data are available for transparency.
